# Graphical modeling and query language for hospitals

**DOI:** 10.1186/2047-2501-1-14

**Published:** 2013-11-04

**Authors:** Janis Barzdins, Juris Barzdins, Edgars Rencis, Agris Sostaks

**Affiliations:** Institute of Mathematics and Computer Science, University of Latvia, Riga, Latvia; Faculty of Medicine, University of Latvia, Riga, Latvia

## Abstract

**Background:**

So far there has been little evidence that implementation of the health information technologies (HIT) is leading to health care cost savings. One of the reasons for this lack of impact by the HIT likely lies in the complexity of the business process ownership in the hospitals. The goal of our research is to develop a business model-based method for hospital use which would allow doctors to retrieve directly the ad-hoc information from various hospital databases.

**Methods:**

We have developed a special domain-specific process modelling language called the MedMod. Formally, we define the MedMod language as a profile on UML Class diagrams, but we also demonstrate it on examples, where we explain the semantics of all its elements informally. Moreover, we have developed the Process Query Language (PQL) that is based on MedMod process definition language. The purpose of PQL is to allow a doctor querying (filtering) runtime data of hospital’s processes described using MedMod.

**Results:**

The MedMod language tries to overcome deficiencies in existing process modeling languages, allowing to specify the loosely-defined sequence of the steps to be performed in the clinical process.

The main advantages of PQL are in two main areas – *usability* and *efficiency*. They are: 1) the view on data through “glasses” of familiar process, 2) the simple and easy-to-perceive means of setting filtering conditions require no more expertise than using spreadsheet applications, 3) the dynamic response to each step in construction of the complete query that shortens the learning curve greatly and reduces the error rate, and 4) the selected means of filtering and data retrieving allows to execute queries in O(n) time regarding the size of the dataset.

**Conclusions:**

We are about to continue developing this project with three further steps. First, we are planning to develop user-friendly graphical editors for the MedMod process modeling and query languages. The second step is to do evaluation of usability the proposed language and tool involving the physicians from several hospitals in Latvia and working with real data from these hospitals. Our third step is to develop an efficient implementation of the query language.

## Background

### Introduction

In 2002, the management professor and renowned author Peter Drucker stated in his book “Managing in the Next Society”, that “health care is the most difficult, chaotic, and complex industry to manage today”, and that the hospital is “altogether the most complex human organization ever devised”. Since then, hospitals have made advances in implementation of the promising health information technologies (HIT) in hopes to achieve major healthcare cost savings, reduce medical errors and improve health outcomes [1]. Unquestionable and measurable has been the positive impact of the HIT on patient safety, quality and continuity of care, and the patient empowerment [2, 3]. So far however there has been little evidence that implementation of the HIT is leading to health care cost savings [3]. One of the reasons for this lack of impact by the HIT likely lies in the complexity of the business process ownership in the hospitals.

While both the management and support processes are directly controlled by the hospital management, the main operational clinical processes which constitute the core value production of the business has generally been owned by the doctors. Since medical professionals and not the managers carry the ultimate responsibility for the patient’s outcomes, the management has a limited control over the doctors’ individual bedside decisions. Therefore, a more profound involvement of the doctors in transforming the processes within their health care organizations has been widely regarded as a factor that is critical for their success [4–7]. This is particularly true considering the fact that up to 85% of all the spending in health care is directly or indirectly controlled by the medical professionals [8].

In contrast to the professional managers who have received an appropriate training and control the administrative resources (e.g., specially dedicated business analysts for extracting process knowledge from the increasing amount of digitally stored data), doctors so far have benefitted to a much lesser degree from these advances in HIT as a tool for better understanding of the patterns and systemic consequences of the clinical decisions they make. The goal of our research is to develop a business model-based method for hospital use which would allow doctors to retrieve directly the ad-hoc information from various hospital databases which is needed in building their process-oriented knowledge for their managerial roles.

For better understanding, we broke down the task of achieving this goal into two steps. First, we developed a new domain-specific language for hospital modeling which allows doctors and managers visualizing the hospital processes. Subsequently, based on this modeling language, we developed an easy-to-perceive graphical query language which permits retrieving specific information needed for the analysis of a particular clinical process. The query language is considered to be the basic added value of this paper. An evaluation of our approach is given in Conclusions.

### Related work

In recent years business processes in hospitals have been studied for the applicability of modeling methods used in other industries. For example, there are published reports of successful usage of BPMN for describing the clinical process for strictly selected group of patients with a specific diagnosis in oncology [9] and the process in selected department for pathology investigations [10]. However, there are also reports suggesting that application of BPMN is difficult in the specific domain of health care, since the nature of health care processes in a multidisciplinary hospital is inherently complex [11], and that has been the basis also for the domain-specific modeling in testing [12].

There are works on querying the descriptions of the business processes without the underlying data, e.g., work of Beeri et.al. [13], where the visual query language BPQL has been introduced, and the BPMN-Q language by Awad [14].

Beeri et.al. [15] went a step further by introducing the BP-Mon – a query language for monitoring business processes, which allows the users defining the monitoring tasks and retrieving their associated reports visually. Although the language is simple enough for IT specialists, it is hardly useable by doctors in the hospitals. For example, the specification of reports (retrieved data) requires knowledge about XML.

Beheshti et.al. [16] introduced a process mining and querying methodology, where data are acquired also from the information system. These data are called *Event Logs* and are grouped into *folder nodes* – a similar concept to *slices* presented in this paper. However, the query language is itself based on SPARQL making it impractical for a broader use by the hospital staff.

## Methods

### Hospital modeling language MedMod

The most-widely known general purpose modeling language UML offers at least three different types of languages, whose elements can be used in hospital modeling.

The first one – UML Activity diagrams (and also the BPMN diagrams) – describes the sequence of activities to be performed. However, this kind of language cannot be directly used in the hospital domain because of the large degree of variations of the order how doctor execute various treatment procedures. The sequence of the activities can only be partially defined here. On the other hand, certain procedures have established protocols and well-defined sequence of activities, e.g. registration of the patient, or anaesthesia used in performing certain types of procedures, and these are suitable for analysis using the UML activity diagram. Therefore, while the activity diagrams can be used in describing some aspects of the hospital operation, they are not applicable for the entire process.

The second type of diagrams are the UML Class diagrams or ontologies (they largely differ in only one aspect – the former uses closed-world semantics, while the latter exploits the benefits of the open-world semantics, e.g., see [17]). This type of diagrams is very convenient for concept modeling, but is not oriented towards modeling the activities. UML allows however perceiving an activity, e.g., X-ray investigation for a patient, as a class. Instances of this class would then be defined as certain X-ray investigations used for specific patients. Further in this paper we will make an essential usage of this type of classes.

The third type of diagrams is UML Use-case diagrams, which combine the elements of the class and activity diagrams. They describe activities called use-cases. It is also stated that use-cases can be perceived as classes, whose instances are concrete executions of these activities. A very useful aspect of the use-case diagrams is their capability for interaction between the use-cases with extending the activity by calling another activity. In other words, the extension point mechanism in the use-case diagrams makes it possible to describe specific control flows having a guard condition (the extension point name), which are executed during the current activity instead of waiting for the activity to complete. This feature isn’t present neither in UML activity diagrams, nor in BPMN, but is very pertinent in the case of hospital modeling. At the same time there are no ordinary control flows in use-case diagrams, because use-case diagrams are a priori dedicated to describing a higher-level functionality.

**Figure 1:**
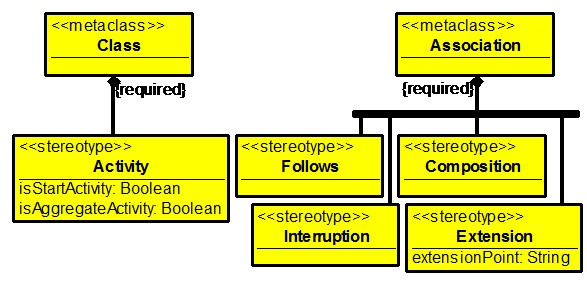
**The UML profile defining the** **MedMod** **language.**

**Figure 2:**
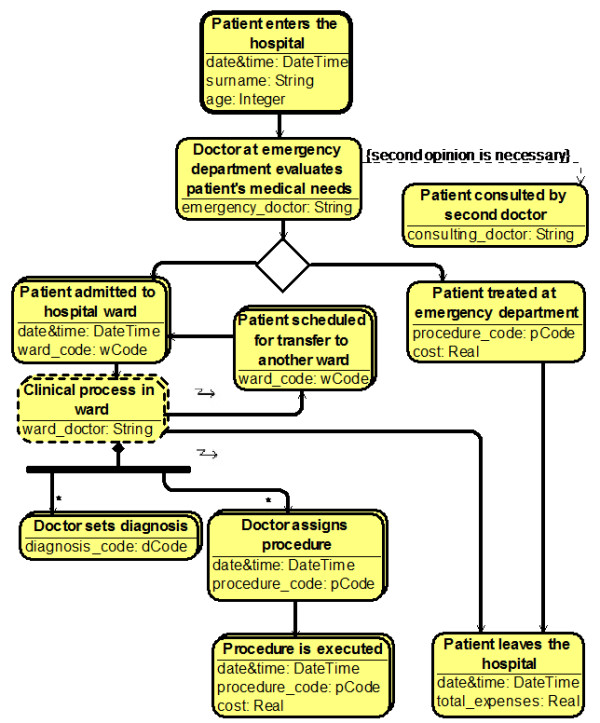
**An example of a** **MedMod** **process.**

This all led us to think that a special domain-specific modeling language is needed for hospital modeling, which would borrow the most useful features from class, activity and use-case diagrams. We have developed such a language called the MedMod. Formally, we can define the MedMod as a profile on UML Class diagrams as can be seen in Figure 1 (OCL constraints defining MedMod more precisely are omitted here). We are however describing the language on examples (see Figure 2) for its easier perception by the domain experts (doctors and managers).

Let us now describe the elements of MedMod in more detail (see Figure 2).

### Activity

Activity is the central element of the MedMod language and denotes a task in time having a start and end moments. Semantically it is related to the Action element of UML Activity diagrams. Examples of Activity are seen in Figure 2 depicted as yellow boxes with rounded corners.

From the linguistic point of view, we divide Activities in three different categories based on how the Activity name is formed. The first type of Activities is the most common one and conforms to the simple present linguistic form – “Doctor sets diagnosis”. The second type of Activities is formed in passive voice and used in cases, when there can be different consequences to some previous Activity leading to execute one of different outgoing flows from it – “Patient admitted to hospital ward”. The third type of Activities refers to a greater process with some given name, which then serves as a name for the Activity – “Clinical process in ward”. These naming conventions are, however, only guidelines for users creating and reading the MedMod diagrams and they are provided for better comprehension of the process. The visual appearance of Activity does not depend on its linguistic type.

Activities can also have attributes of five primitive data types – Integer, Real, String, Boolean and DateTime. These attributes can be specified for every concrete Activity at diagram creation, and different values can be assigned to these attributes of concrete instances of the Activity at run-time. Since there are very detailed codificators in the medical world for coding every procedure, diagnosis or other attributes (see Health Level Seven International, the global authority on standards for interoperability of health information technology [18]), we also allow using enumerations as data types. For instance, the Activity “Doctor assigns procedure” has an attribute “procedure_code”, whose values come from the enumeration “pCode” (see Figure 2).

Exactly one of the Activities of every MedMod diagram is denoted as the Master Activity meaning that the execution of the diagram starts with this Activity (there can be no ingoing arrows to this Activity). Master Activity has a slightly different visualization – a bolder frame (see Activity “Patient enters the hospital” in Figure 2).

### Follows

This type of oriented relation can be established between two Activities A and B meaning that Activity B can only start after Activity A has ended (the same semantics as the Control Flow of UML Activity diagrams). It is allowed for several Activities to follow the same Activity – the XOR semantics is implied in this case meaning that only one of those outgoing flows can be executed. We denote this situation by introducing a new diamond-shaped graphical element seen in Figure 2. It is also allowed to have several ingoing flows into an Activity implying the OR semantics, i.e., the following Activity can start executing when at least one flow has executed, and several instances of that Activity can arise, if several incoming flows executes at different times. It is, however, not allowed to introduce several parallel outgoing flows from the same Activity. We substitute the parallel branching of UML Activities with a more general feature, the composition, by introducing so called Aggregate Activities and their parts – Component Activities that can be executed simultaneously.

### Composition

A composition between two Activities can be established, if one Activity (called the Aggregate) semantically consists of one or more other Activities (called the Components). It has an analogy with the relation “includes” of UML Use-case diagrams. We have borrowed the notation for the Aggregate part of Composition (the filled diamond) from the UML Class diagrams. Also, a composition fork graphical element can be introduced to collect the Components of the same Aggregate Activity (seen in Figure 2). For instance, Activity “Clinical process in ward” consists of two types of Activities – “Doctor assigns procedure” and “Doctor sets diagnosis” (notice a slightly different visualization – a dashed frame – for Aggregate Activities in Figure 2). Each Component Activity can appear several times within the Aggregate, therefore we also allow cardinalities to be attached to the Component end of a Composition (the default cardinality is 1).

### Interruption

An interesting phenomenon relates the composition – what is the semantics of a Follows-type relation going out from the Aggregate Activity? It was stated before that the Follows flow can execute when the Activity A has ended. But the Aggregate Activity can actually never end, if it has at least one Component having a cardinality, e.g., * (many). In this case the Aggregate is constantly waiting for new and new Component instances to born, and only some force from outside can decide, when to stop the waiting process. We must therefore introduce a new type of control flow – an Interruption – stating that if there is an outgoing Interruption flow from the Aggregate Activity A to some Activity B, it means that the Activity A is suspended, when the flow is executed (i.e., when the Activity B needs to be started) meaning that it can no more create new Component instances (already created Component instances continues to execute normally). For instance, in Figure 2 the Activity “Clinical process in ward” is suspended when the doctor decides to either transfer the patient to another ward, or to discharge the patient. The Interruption flow is adorned with a jagged “lightning bolt” arrow. Simple Activities can also be interrupted in similar manner.

### Extension

Extension is an oriented relation between two Activities A and B meaning that Activity B can be called at some time during the execution of Activity A. This feature allowing us to extend the Activity is also borrowed from UML Use-case diagrams. The call is triggered, when some predefined condition occurs. The condition is described as an Extension point name and attached to the Extension. For instance, a doctor can decide that a “second opinion is necessary” (the Extension point name) during the evaluation of patient’s medical needs. In that case another Activity “Patient consulted by second doctor” is called (see Figure 2).

Using the four abovementioned elements, one can define a MedMod process serving at least two purposes: 1) the visualization of hospital processes can help doctors and management of the hospital in performing their daily tasks better; 2) one can use the graphical process in order to perform queries on their underlying real data. This is one of the added values of this paper. To achieve the second part of the goal stated in the Introduction, we must first introduce a new concept of a slice being exploited in the next section. If we look at the MedMod diagram from the process point of view, we can notice that every instance of the Master Activity define a separate transaction consisting of those instances of Activities that can be reached from the instance of the Master Activity (these are called run-time instances in the process modeling world). We call the set of all run-time instances within a transaction a slice. The basic assumption we make here is that no two slices can ever share any common instances. It must be notices that certain Activities can have several instances within a slice (because of loops and cardinalities of type “many”). We use a slightly different visual representation for this type of Activities for better perception as can be seen in Figure 2.

When the process is described, is it very important for the doctor to be able to see the run-time instances (both within a slice and over several slices) with their respective attribute values from different points of view. One idea here could be to export all slices over some period of time to Microsoft Excel and then use its features to analyse the data. The main problems here arise from the fact that we can have loops and cardinalities of type “many” allowing several run-time instances appear for a concrete Activity. Developing a non-trivial query for this case may involve serious “Excel programming” not being possible for a doctor. To overcome this problem, we have developed a simple process query language that is based on the process diagram that needs to be analysed.

### Process query language

The Process Query Language (PQL) has been based on MedMod process definition language. The purpose of PQL is to allow a doctor interested in clinical processes querying (filtering) runtime data of hospital’s processes described using MedMod. In fact, a doctor should be able to ask the ad-hoc questions like “*How much did the Dr. Jekyll’s patients cost?*” or “*Which patients with Pneumonia had more than two X-rays?*”. This paper describes general ideas behind PQL and does not touch any implementation details except the efficiency of query execution. We assume that technical problems, like the import of runtime data from hospitals information system to MedMod data structures, have been already solved.

**Figure 3:**
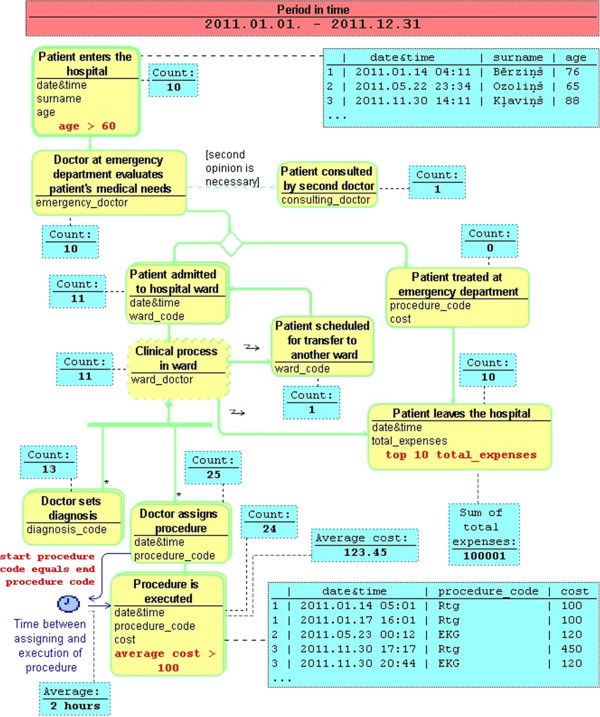
**An example of Process Query Diagram describing a hospital operation.**

Asking questions begins with choosing (opening) the MedMod process diagram, which describes the process under inspection, switching to the **filtering mode** (for example by pressing on a special toolbar button) and setting the time interval the doctor is interested in. As a result, a new diagram – *Process Query Diagram* – is created. It contains the chosen process description in the MedMod syntax. In addition, every activity node in the diagram has an indicator (the attached box) showing the number of instances in *the initial dataset* – all slices corresponding to the chosen time interval (see Figure 3 – an example where the details described in this section can be viewed).

**Figure 4:**
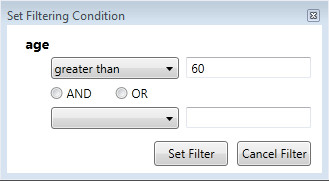
**Filtering condition input form – comparison operation on attribute.**

Now the doctor can undertake two types of actions – she can **set filtering conditions** or **retrieve data**. Setting filtering conditions can be initiated by selecting an action node. Typically, the doctor can choose to set a filter on attribute values of the node. The attribute can be selected, for example, by clicking on it. There are several options for filtering. The first filtering option is **the comparison operations** like *equals*, *greater than*, *less than*, *contains*, *begins with*, etc. The actual list of operations depends on the data type of the attribute. The same principle has been used in spreadsheet applications like *Microsoft Excel* for setting simple filtering conditions on column values. The typical filter input form has been shown in Figure 4.

First, the comparison operation is selected (e.g., *greater than*). Second, a value is given. If the possible values can be retrieved from the fixed list (e.g., HL7 codes or doctors of the hospital), then the input form offers a list (e.g., via a combo box) the user can choose from. Following the simplicity of spreadsheet applications, only one extra comparison operation is allowed here. User may choose one of the following options – either both conditions are mandatory (logical AND), or at least one of the conditions must be met (logical OR). Thus, most of the typical conditions, including value intervals, can be given using such input form. In the process query diagram a filtering condition appears as a label in the corresponding activity node. Thus, the doctor is always aware of filtering conditions that have been set. Immediately after the filtering condition has been created or updated, it is applied on the dataset. The filtered dataset contains all instances from those slices, which contains instances conforming to the filtering condition. As a consequence, all data displayed in the diagram (e.g., the indicators of number of instances) are updated.

**Figure 5:**
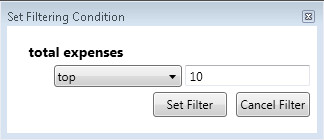
**Filtering condition input form – data partitioning operation.**

The second filtering option is **the data partitioning operations** like getting *Top* or *Bottom* instances based on some attribute. Doctor may ask for 10 slices, where *total expenses* are the largest. She should select the corresponding activity and choose the data partitioning option. The filter input form is shown in Figure 5.

First, the partitioning operation is selected (e.g., *Top*). Second, a value is given. When the filter is applied, the filtered dataset contains only instances from those slices, which contain instances with ten largest *total expenses* values. It should be noted that partitioning operations are applied on the dataset retrieved by applying the comparison operations. If several data partitioning operations have been set, then the result is the intersection of slices retrieved by partitioning operations.

If there is a possibility that a slice may contain more than one instance of the same type (e.g., if there is a composition with a cardinality “many” or flows heading backwards), then it is possible to set a filtering condition on **aggregate functions**. The filtering conditions may be applied to the *Sum* or *Average* of attribute values of instances in the slice. The filtered dataset contains all instances from those slices, which contains instances of the filtered type having sum or average value of the given attribute within values specified by the condition. For example, the doctor may ask for those slices, where average *cost* of “*Procedure is executed*” is greater than 100. Another option is to set comparison condition on number of instances within a slice using the *Count* operation. For example, doctor can ask for slices where “*Doctor sets diagnosis*” happened more than once. Setting this condition may be initiated by clicking on the action node itself (not on an attribute).

As stated before, there are two types of querying actions – setting filtering conditions and retrieving data. The former is explained above. Now let us proceed to the latter. Although the MedMod diagram elements cannot be modified in PQL, user may supplement the diagram with additional details. The possible options appear in the palette and can be dragged into the diagram, thus retrieving data within the diagram.

The first option is **the time interval**. It can be drawn between actions containing *date&time* attribute. The time interval symbol appears in the diagram. The incoming arrow denotes interval’s start activity and the outgoing arrow denotes interval’s end activity. The interval has also a name. In fact, we may think of a time interval as of a derived attribute in the master activity of the slice, which is computed as the difference between interval’s end activity *date&time* and interval’s start activity *date&time*. Note that there may exist multiple interval values because of multiple start and end instances within the slice. To specify more precisely the instances the time interval should be measured between, a conditional expression may be used. For example, if the doctor wants to measure time between “*Doctor sets procedure*” and “*Procedure is executed*” for those instances, whose *procedure code* matches, she should supply the conditional expression stating “*start procedure code equals end procedure code*”. If no conditional expression has been supplied, then the interval between two adjacent (in time) instances of corresponding types is measured. Once the time interval has been defined, it can be used in filtering conditions.

An important feature is the possibility to add intervals between instances allocated in different slices, e.g., between patient’s multiple appearances in the hospital. It would require **grouping of slices** to be introduced. Grouping would allow merging slices depending of some attribute values, e.g. patient’s *surname*.

The second data retrieving option is the set of **aggregate functions**, which can be evaluated over the filtered dataset in order to obtain a single number as an answer to the question asked. They are: **Count**, **Sum** and **Average** meaning respectively the number of instances within the dataset, the sum of the given attribute values over all instances in the dataset and the average of the given attribute values over all instances. They can be applied by dragging the selected function from the palette to the corresponding activity node (for *Count*) or to the attribute (for *Sum* and *Average*). The result (one number) appears on the diagram as an indicator box, which displays the computed value. In fact, the number of instances of each activity appears in the diagram by default. However, they can be also removed from the diagram.

The third possible option of retrieving data is a **list of all instances** corresponding to the selected activity. Dragging corresponding palette element to an activity initiates the display of all instances of corresponding type in the filtered (or initial if no filtering conditions are applied) data set. They are displayed as a table, where each row represents an instance and columns represent the attribute values. There is also one special column containing slice’s ID the instance belongs to. Since it is possible to display several *instance tables* at once, the presence of an ID in each of them helps to recognize data from the same slice across several tables. If the filtering conditions have been changed, then the content of all tables and indicator boxes is recomputed.

Thus, the basic steps in querying are:

1. Process Query Diagram is created from a MedMod diagram – the initial dataset is determined by initial time interval given by the doctor and the indicator boxes denoting the number of instances for each action appear;
2. Doctor may apply two types of filtering conditions – comparison operations on attributes and aggregation functions or data partitioning operations;
3. Doctor may retrieve data into the diagram – aggregate values (Count, Sum, Average), which are one number answers, or instance tables;
4. Changing filtering conditions immediately reflects on displayed data.

## Results and discussion

Since the main target auditory of the PQL is non-IT professionals, we have to assess the adequacy of the query language for its purpose – effective ad-hoc querying of hospital’s data. We will discuss two *areas of effectiveness* –usability of language and efficiency of query execution.

### Usability of the query language

To test the practical aspects of using the PQL we presented it to a group of seven doctors working in a hospital. Our primary interest was to assess the “readability” of the designed clinical process model and of the information filtered with its application by the end-users. After a short instruction about the syntax of process description, available filtering mechanisms and visualizing the retrieved information in data indicator boxes next to each of activity nodes, doctors were asked to explain the meaning of the three prepared screenshots representing retrieved data with a use of the query language. All participants of this test demonstrated that they could accurately retrieve the question to be asked by applying the proposed querying techniques in our hospital model. In general, all of the participating doctors rated the presented methodology positively and noted not only the potential for this tool to facilitate management and improve the transparency of clinical processes, but also its potential for research on the impact that certain variables have on the treatment outcomes.

The evaluation of the language *in action* – the evaluation of the usability for the proposed method of building queries can’t be done properly without the implementation of the language. However we are aware that the direct manipulation interaction mechanism [19] used by the PQL (the dynamic response to each step in construction of the complete query), which allows the physician to see immediate reaction to his every action, shortens the learning curve greatly and reduces the number of errors in the process of building queries. For example, the physician wants to get information about patients having diagnosis *Chickenpox*, which is a typical childhood disease. In order to do that, he would set a simple condition on activity *Doctor sets diagnosis* (see Figure 3). Number of patients matching the condition would be retrieved and shown immediately. Now if the physician wants to refine the query and ask about a certain age group, e.g., patients elder than 6 years, but accidentally puts a number 67 instead of 6 in the condition, he will most likely get no data matching the condition or some small number of patients. Thus, the physician may immediately cancel the last condition using, for example, *Undo* button, and get to the previous state.

It may seem that simple means of querying described in the previous sections can answer just simple questions, and it is true. However combining simple answers may give answers to more complicated questions. Let’s take a simple question – “*How many patients had diagnosis Pneumonia?*” The answer is a single number – number of patients with diagnosis Pneumonia. Now adding the second condition, e.g., *“How many patients had diagnosis Stroke?”* answers the conjunction of both questions. “*How many patients had diagnosis Pneumonia and Stroke*?” Thus we obtain another number – number of patients with both diagnoses. If we have remembered the first answer, then we can interpret the sequence of answers as an implication, e.g., “*If a patient has Pneumonia, then there is a probability****p****that he has Stroke*”, where *p* is the number obtained by dividing the second result (number of patients with Stroke and Pneumonia) by the first result (number of patients with Pneumonia only). In fact, the data mining task, association rule learning, like market basket analysis (“*if the customer has bought A, then there is a probability p that he will buy B”*), is being solved here. However unlike in the classical data mining where new hypotheses about data are extracted automatically [20], here the hypotheses generated by human intelligence can be verified and it can be done very efficiently – in linear time regarding size of the dataset (see the next section). Automatic extraction of hypotheses requires non-linear (exponential) time regarding to the number of *items* - possible attribute values of *interesting* MEDMOD activities [21].

### Efficiency of query execution

The efficiency of the query execution has been an important aspect of implementation of query languages, because of obvious reasons - the result of query have to be obtained in reasonable time. It is conventional that the linear time of execution of an algorithm regarding the size of data is acceptable. Now let us show that the execution of PQL queries (using most of typical constructs) is **linear** regarding the number of instances in the initial dataset.

As it was mentioned before, building PQL queries has two main actions - filtering and retrieving data. Let’s discuss complexity of the filtering in PQL. One of the filtering options was setting simple conditions (comparison operations) on attribute values. The set of possible comparison operations has been chosen in such way that evaluation of condition on a single instance of the dataset can be performed in constant time. The reason is the simplicity of the allowed operations – there are no references allowed to other instances. Of course, the constant depends on the implementation of objects and the size of used MedMod diagram. The evaluation of a single condition on the complete dataset can be done by evaluating the condition on every instance in the dataset. If the instance meets the condition, then the corresponding slice should be included into the filtered dataset. The following pseudo code illustrates the evaluation of a condition:

**function***evaluateCondition(initial_dataset, condition)*

*filtered_dataset* :**=** Ø

**foreach***slice***in***initial_dataset***do**

**foreach***instance***in***slice***do**

**if***condition(instance)==true***then**

**add***slice***to***filtered_dataset*

**exit loop**

**return***filtered_dataset*

The main idea is to go through all slices in the initial dataset and check all instances in the particular slice. If the condition evaluates to *true* on the instance, then the slice is added to the filtered dataset. It is easy to see that in the worst case all instances in the initial dataset have to be checked to evaluate the condition, but no more, because slices are non-overlapping. However, checking a single instance does not require more time than some constant, thus **the total time needed to evaluate a single condition on the initial dataset is O(*n)*, where*n*is the number of instances in the dataset.**

It should be noted that the second filtering option, data partitioning operation, like, getting *k Top* or *Bottom* slices depending on some attribute, requires no more than *k* inspections of every instance. The idea is to maintain the list of *k* top or bottom instances. Every new instance under inspection should be compared to at most every instance in the list. Since typically *k* is much smaller than the total size of the data set (it is meaningful to query just for few extremes of the kind), we have restricted the possible inputs of k, thus the evaluation of data partitioning operations can be done also in O(n) time, where n is the size of the data set (keeping in mind the multiplicative constant *k*). The third filtering option was comparison to aggregates of attribute values within a slice, like, *Avarage* or *Sum*. It is easy to see that computing aggregates also can be done in the linear time.

Thus, the complete execution of filtering step can be described by the following pseudo code:

**function***executeFiltering(initial_dataset, conditions)*

*filtered_dataset***:=***initial_dataset*

**foreach***condition***in***conditions***do**

*filtered_dataset***:=***evaluateCondition(filtered_ dataset, condition)*

**return***filtered_dataset*

It is easy to see that the execution of filtering step in PQL has linear time complexity regarding size of the dataset.

Now let’s discuss the complexity of data retrieving operations. The simplest results that can be obtained in PQL are the single number answers. They are aggregate functions *Count*, *Sum* and *Average* over the filtered dataset. As we have discussed above, aggregates can be computed in time O(n), where n is the size of the dataset. The same linear time is needed also to get the list of all instances that correspond to the given MedMod activity – every instance in the dataset must be checked just once.

It should be noted that the prototype of PQL graphical editor has been implemented and it includes also an experimental implementation of query execution without any optimizations. The implementation is meant to serve for the usability testing of the language on small datasets. The size of the initial dataset used in the experiment was 5000 slices (~64 000 instances). On the Intel® Core™ i7-3610QM CPU @ 2.30 GHz, 8 GB RAM, Windows 7 64-bit operating system workstation queries executed in less than 1 second (the time needed to filter, retrieve and display data). Based on the results of the experimentations we believe, that an optimized solution would work acceptably also for larger datasets, e.g., 30000 slice dataset, that corresponds to the number of patients treated in an average hospital in Latvia (500 beds).

### Summary

The main advantages of the PQL are: 1) the view on data through “glasses” of familiar process, 2) the simple and easy-to-perceive means of setting filtering conditions require no more expertise than using spreadsheet applications (like *MS Excel*), 3) the dynamic response to each step in construction of the complete query, that allows the doctor to see the immediate reaction to every action - it shortens the learning curve greatly and reduces the error rate, and 4) the selected means of filtering and data retrieving allows to execute queries in O(n) time regarding the size of the dataset (number of activity instances).

As a drawback of the proposed query language should be mentioned the need to import the data from the hospital’s information system to the MedMod data structures. What is important, the import should be adjusted for every change in the real-world process and this problem has not been researched yet.

## Conclusions

We are about to continue developing this project with three further steps. First, we are planning to develop user-friendly graphical editors for the MedMod process modeling and query languages. We have already built prototypes of these editors, which were used in creating the proof of concept, e.g., examples seen in Figures of this paper. Nowadays, it is a rather easy task to develop graphical editors for such domain-specific languages within some of the tool building platforms like GRAF [22] or METAclipse [23].

The second step is to do evaluation of usability the proposed language and tool involving the physicians from several hospitals in Latvia and working with real data from these hospitals. Thus, also missing part of the research - integration with hospital’s information systems is to be researched.

Our third plan is to develop an effective implementation of the query language. Success of the PQL depends mainly on the efficient implementation of the query execution. This task, is closely related to the pattern matching problem [24] in the field of implementation of model transformation languages (like, MOLA [25], lQuery [26], etc.), which have already been used in the various areas of Model-Driven Engineering.
